# The Physiological Response of Lettuce to Red and Blue Light Dynamics Over Different Photoperiods

**DOI:** 10.3389/fpls.2020.610174

**Published:** 2021-02-12

**Authors:** Giedrė Samuolienė, Akvilė Viršilė, Jurga Miliauskienė, Perttu J. Haimi, Kristina Laužikė, Aušra Brazaitytė, Pavelas Duchovskis

**Affiliations:** Lithuanian Research Centre for Agriculture and Forestry, Institute of Horticulture, Babtai, Lithuania

**Keywords:** xanthophylls, carotenes, soluble sugars, organic acids, antioxidant activity, photosynthesis, dynamic light, photoperiod

## Abstract

This study aimed to evaluate the effect of dynamic red and blue light parameters on the physiological responses and key metabolites in lettuce and also the subsequent impact of varying light spectra on nutritive value. We explored the metabolic changes in carotenes, xanthophylls, soluble sugars, organic acids, and antioxidants; the response of photosynthetic indices [photosynthetic (Pr) and transpiration (Tr) rates]; and the intracellular to ambient CO_2_ concentration ratios (*C*_*i*_/*C*_*a*_) in lettuce (*Lactuca sativa* L. “Lobjoits Green Cos”). They were cultivated under constant (con) or parabolic (dyn) blue (B, 452 nm) and/or red (R, 662 nm) light-emitting diode (LED) photosynthetic photon flux densities (PPFDs) at 12, 16, and 20 h photoperiods, maintaining consistent daily light integrals (DLIs) for each light component in all treatments, at 2.3 and 9.2 mol m^–2^ per day for blue and red light, respectively. The obtained results and principal component analysis (PCA) confirmed a significant impact of the light spectrum, photoperiod, and parabolic profiles of PPFD on the physiological response of lettuce. The 16 h photoperiod resulted in significantly higher content of xanthophylls (neoxanthin, violaxanthin, lutein, and zeaxanthin) in lettuce leaves under both constant and parabolic blue light treatments (BconRdyn 16 h and BdynRdyn 16 h, respectively). Lower PPFD levels under a 20 h photoperiod (BdynRdyn 20 h) as well as higher PPFD levels under a 12 h photoperiod (BdynRdyn 12 h) had a pronounced impact on leaf gas exchange indices (Pr, Tr, *C*_*i*_/*C*_*a*_), xanthophylls, soluble sugar contents, and antioxidant properties of lettuce leaves. The parabolic PPFD lighting profile over a 16 h photoperiod (BdynRdyn 16 h) led to a significant decrease in *C*_*i*_/*C*_*a*_, which resulted in decreased Pr and Tr, compared with constant blue or red light treatments with the same photoperiod (BconRdyn and BdynRcon 16 h). Additionally, constant blue lighting produced higher α + β-carotene and anthocyanin (ARI) content and increased carotenoid to chlorophyll ratio (CRI) but decreased biomass accumulation and antioxidant activity.

## Introduction

The effects of light on plants are complex, as it controls photosynthesis, plant growth, and developmental processes. Plants generally absorb radiation in the visible electromagnetic spectrum; however, not the entire spectrum of light is beneficial (Hasan et al., 2017). Under controlled environmental conditions, the mixed application of red and blue light-emitting diodes (LEDs) is usually the first choice where plant growth is a priority (Shen et al., 2014; Bantis et al., 2018). Red and blue LEDs can act as a principal light source, with the potential to improve photosynthesis by stimulating the stomatal activity, which can enhance dry mass and yield (Sabzalian et al., 2014). Various studies have demonstrated that controlled amounts or specific spectra of light affect photosynthetic characteristics (Dong et al., 2014; Samuolienė et al., 2020), metabolism (Samuolienė et al., 2013; Lee et al., 2016), and antioxidant properties (Samuolienė et al., 2012; Lekkham et al., 2016). However, these experiments are typically performed in controlled environment chambers with constant irradiance during the day and abrupt transitions between light and dark at dawn and dusk. In natural environments, plants are adapted to diurnal fluctuations in irradiance and light quality, with gradual shifts between light and dark at the beginning and the end of the day. Moreover, the light in controlled environment chambers differs from natural sunlight in several ways, including qualitative spectral and quantitative intensity changes over the course of the day (Annunziata et al., 2017).

The light-dependent reactions of photosynthesis involve electron and proton transfer from water to NADP^+^ to form NADPH, whereas the dark-dependent reactions involve the fixation of CO_2_ into carbohydrates *via* the Calvin–Benson cycle, regenerating ADP and NADP^+^ (Johnson, 2016). Concentrations of Mg^2+^, pH, and thioredoxin play a key role in regulating the activity of the Calvin–Benson cycle enzymes, ensuring that the activity of the light-dependent and light-independent reactions are closely coordinated. Pigment molecules (chlorophylls *a* and *b*, β-carotene, zeaxanthin, neoxanthin, violaxanthin, and lutein), located in the thylakoid membrane, absorbing red and blue light, induce the process of photosynthesis. Moderate stress caused by different environmental factors, including light, influences the metabolism of carotenoids (Fanciullino et al., 2014), as some carotenoids play a key role in the dissipation of excess absorbed energy through the xanthophyll cycle. In light-collecting antennae, carotenoids absorb light in the blue and green region, transferring the absorbed energy to the chlorophylls, thus increase photosynthetic efficiency by expanding the range of wavelengths absorbed (Mozzo et al., 2008; García-Plazaola et al., 2012). Furthermore, exposed to excessive radiation, xanthophylls quench the singlet excited state of chlorophyll in photosystem II (Robert et al., 2004). This process, named non-photochemical quenching, is linked to the xanthophyll cycle (García-Plazaola et al., 2012); preventing photodamage, it allows plants to acclimate to variable levels of light. Additionally, many carotenoids act as antioxidants. For instance, zeaxanthin can protect membrane surfaces from hydrophilic oxidants (Johnson, 2016), Xanthophylls are precursors of abscisic acid, which modulates stress and developmental processes (Chernys and Zeevaart, 2000), and by quenching excited chlorophyll molecules, β-carotene exerts an effective photoprotective action (Johnson, 2016).

Plants rely on both rapid response and delayed mechanisms to dynamically acclimate to their environment. Immediate and sensitive responses involve redox and ROS, while delayed responses are related to the accumulation of sugars which can reduce photoinhibition and the associated production of ROS in photosynthetic tissues (Fanciullino et al., 2014). The overall parameter intensities from both response types are integrated to realize appropriate long-term acclimation (Dietz, 2015). Sugars, as the end products of photosynthesis (Stein and Granot, 2019), are information-rich signaling molecules involved in the mechanisms that balance carbohydrate supply and usage in growth and storage processes. For instance, glucose coordinates internal sugar signals with external light conditions (Moore et al., 2003). There are several sugar-sensing mechanisms, including sensory systems for hexoses, sucrose, and raffinose, with increasing evidence suggesting that sugar contents are monitored in the plastids (Häusler et al., 2014). Additionally, photosynthesizing cell metabolisms respond quickly to changes in photosynthetic conditions, in particular, light, temperature, or CO_2_ availability, which in turn depends on the state of stomatal opening (Dietz, 2015). However, contradictory data on the effect of soluble sugars on the synthesis of carotenoids reflect the distinct strategies in the leaves as a function of light influence (Urban and Alphonsout, 2007). The former leads to a benefit of carotenoids to increase protection against photodamage, leading to a loss of carotenoids when senescence is triggered (Fanciullino et al., 2014).

The aim of this study was to evaluate the effect of dynamic red and blue light parameters on lettuce physiological response and metabolite contents and thereby its impact on nutritive value.

## Materials and Methods

### Growing Conditions

Lettuce (*Lactuca sativa* L. cv. “Lobjoits Green Cos”) was grown in a growth chamber in peat substrate (Profile 1, JSC Durpeta, Lithuania) (pH 6, accuracy ± 0.01 pH units). The average concentrations of nutrients (mg L^–1^) in the substrate were as follows: N, 110; P_2_O_5_, 50; and K_2_O, 160. The microelements Fe, Mn, Cu, B, Mo, and Zn were also present. Electrical conductivity (EC) varied between 1.0 and 2.5 mS cm^–1^ (±0.03 m). One seed was seeded into a 120 ml vessel, and 28 plants for each treatment were analyzed. Plants were watered as needed. Experiments were performed in a walk-in controlled environment growth chamber (4 × 6 m). Day/night temperatures of +21/17 ± 2°C were established, and the relative humidity was maintained at 50–60%.

**FIGURE 1:**
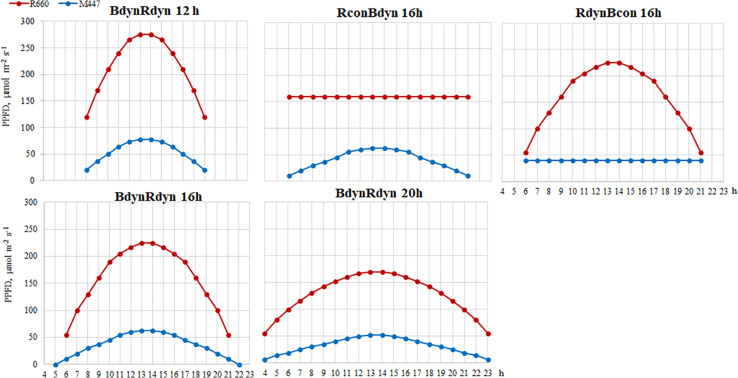
Experimental setup of parabolic and constant light profiles. Photosynthetic photon flux densities (PPFDs) and photoperiods of the applied light-emitting diode (LED) spectra. B, blue, 452 nm; R, red, 662 nm. Total PPFD, 200 μmol m^–2^ s^–1^ which corresponds to daily light integral (DLI) of 11.5 m^–2^ per day.

LED fixtures, consisting of commercially available LEDs with emission wavelengths (λ) of blue (λ = 452 nm, LedEngin LZ1-00B200, Osram Sylvania, United States) and red (λ = 662 nm, Luxeon Rebel LXM3-PD01-0300, Lumileds, United States), were used for lettuce lighting. Plants grew under constant (con) or parabolic (dyn) blue (B) and/or red (R) LED photosynthetic photon flux density (PPFD) at 12, 16, and 20 h photoperiods, but the same daily light integral (DLI; 2.3 and 9.2 mol m^–2^ per day for B and R) was maintained for each light component in all treatments (Figure 1). PPFD was measured and regulated at the vessel level by using a photometer–radiometer (RF-100, Sonopan, Poland).

### Determination of Pigments

Xanthophylls (neoxanthin, violaxanthin, lutein, and zeaxanthin), carotenes (carotenes α and β), and chlorophylls (chlorophylls *a* and *b*) were extracted using 80% acetone (1 g of sample ground with liquid N_2_ and 10 ml of solvent), centrifuged (5 min, 4,000 rpm), and filtered through a 0.45-μm nylon membrane syringe filter (VWR International, United States). The contents of carotenes, xanthophylls, and chlorophylls were evaluated using a Shimadzu HPLC (Japan) instrument equipped with a diode array detector (SPD-M 10A VP) on a YMC Carotenoid column (3 μm particle size, 150 × 4.0 mm) (YMC, Japan). The mobile phase consisted of A (80% methanol, 20% water) and B (100% ethyl acetate). The gradient was as follows: 0 min; 20% B, 2.5 min; 22.5% B, 20–22.5 min; 50% B, 24–26 min; 80% B, 31–34 min; 100% B, 42–47 min; and 20% B, flow rate 1 ml min^–1^ (Edelenbos et al., 2001). The diode array detector was employed at 440 nm, and the absorption spectra of xanthophylls, carotenes, and chlorophylls were identified using an external standard calibration method.

### Determination of Sugars

About 0.5 g of fresh plant tissue was ground and diluted with deionized H_2_O. The extraction was carried out for 4 h at room temperature with mixing. Samples were centrifuged at 14,000*g* for 15 min. A clean-up step to remove soluble proteins (Brons and Olieman, 1983) was performed prior to the chromatographic analysis. Briefly, 1 ml of the supernatant was mixed with 1 ml 0.01% (w/v) ammonium acetate in acetonitrile and incubated for 30 min at +4°C. The samples were centrifuged at 14,000*g* for 15 min and filtered through a 0.22 μm PTPE syringe filter (VWR International, United States). Sugars were analyzed according to Ma et al. (2014) with modifications. The analyses were performed on a Shimadzu HPLC (Japan) instrument equipped with an evaporative light scattering detector (ELSD). Separation of fructose, glucose, sucrose, and maltose was performed on a Shodex VG-50 4D HPLC column with deionized water (mobile phase A) and acetonitrile (mobile phase B) gradient. The gradient was maintained at 88% B for 13 min, changed linearly to 70% B in 9 min, kept at 70% B for 1 min, and raised back to 88% B for 2 min, and the column was equilibrated to 88% B for 5 min. The flow rate was 0.8 ml min^–1^.

### Determination of Organic Acids

Approximately 0.5 g of fresh plant tissue was homogenized and diluted with deionized H_2_O (1:10) (w/v) and heated in a water bath for 30 min at +50°C. The extract was centrifuged at 10,000 rpm for 15 min and filtered through a 0.22 μm PTPE syringe filter (VWR International, United States). Organic acids were analyzed according to Wang et al. (2014) with modifications. The analyses were performed on a Shimadzu HPLC (Japan) instrument equipped with a refractive index detector. Separation of oxalic, oxaloacetic, malic, ascorbic, folic, citric, succinic, and fumaric acids was performed on a C18 column (4.6 mm × 250 mm, 5 μm) (Nucleodur) with 0.05 M sulfuric acid in deionized water isocratic elution. The flow rate was 0.5 ml min^–1^. The peak was detected at 210 nm and identified using an external calibration method.

### Determination of Antioxidant Activity

The antioxidant activity of lettuce leaves was evaluated using DPPH (2-diphenyl-1-picrylhydrazyl), ABTS [2,2′-azino-bis (3-ethylbenzothiazoline-6-sulfonic acid) diammonium salt], and an Fe^2+^ reducing antioxidant power (FRAP) assay (Kraujalytė et al., 2013). Extracts were prepared by grinding the plant material with liquid nitrogen and dilution with 80% methanol 1:10 (w/v). After 24 h, the extracts were filtered through cellulose filters.

The DPPH free radical scavenging activity was determined by mixing a diluted extract with 0.06 M methanolic DPPH solution and monitoring radical quenching every minute for 16 min to measure the absorbance at 515 nm (M501, Camspec, United Kingdom). The results are presented as DPPH free radical scavenging activity, μmol g^–1^ of fresh plant weight.

The ABTS radical solution was prepared by mixing 50 ml of 2 mM ABTS with 200 μl 70 mM K_2_S_2_O_8_, allowing the mixture to stand in the dark at room temperature for 16 h before use. The working solution was diluted to obtain an initial absorbance of AU 0.700 at 734 nm (M501, Camspec, United Kingdom); 100 μl of the samples were mixed with 2 ml ABTS solution and absorbance was monitored for 11 min. The results are presented as ABTS free radical scavenging activity, μmol g^–1^ of fresh plant weight.

For the FRAP assay, the working reagent was prepared by mixing acetate buffer (300 mM, pH 3.6), a solution of 10 mM 2,4,6-tripyridyl-s-triazine (TPTZ) in 40 mM HCl, and 20 mM FeCl_3_ × 6H_2_O at 10:1:1 (v/v/v). Twenty microliters of the sample was mixed with 3 ml of working solution and incubated in the dark for 30 min. Then, the absorbance at 593 nm was read. The antioxidant power was expressed as Trolox equivalent antioxidant capacity (TEAC, μmol Trolox per g^–1^ of fresh plant weight) and Fe^2+^ antioxidant capacity (Fe^2+^ μmol g^–1^ of fresh plant weight).

### Leaf Gas Exchange Indices

Photosynthetic rate (Pr, μmol CO_2_ m^–2^ s^–1^), transpiration rate (Tr, mmol H_2_O m^–2^ s^–1^), and the intercellular to ambient CO_2_ concentration ratio (*C*_*i*_/*C*_*a*_) were measured on the third developed leaf, using a portable photosynthesis system (LI-COR 6400XT, United States) under the conditions at +21°C, with a CO_2_ concentration of 400 μmol mol^–1^ and 60% relative humidity. Artificial irradiation was supplied to the leaf using different LEDs (665 and 470 nm), but their respective intensities were the same for PPFD 1,000 μmol m^–2^ s^–1^. Photosynthesis was measured from 9 to 12 a.m.

### Spectral Reflectance Indices

Spectral reflectance was measured using a leaf spectrometer (CID Bio-Science, United States) from 9 to 12 a.m. Reflection spectra obtained from the leaves were used to calculate the carotenoid reflectance index (CRI) and the anthocyanin reflectance index (ARI).

CRI, which shows carotenoid to chlorophyll ratio, was evaluated using the following formula:

(1) CRI=(1/ρ510)-(1/ρ700)

ARI evaluates changes in anthocyanin amount:

(2) ARI=(1/ρ550)-(1/ρ700)

where ρ510, ρ550, and ρ570 represent the leaf reflectance integrated over a 10 nm wavelength band centered on 510, 550, and 570 nm, respectively.

### Biometric Measurements

At the end of the experiment, plant fresh mass (FW) was determined by harvesting leaves from five different plants per light treatment. Leaf area (LA) was determined using a leaf area meter (AT Delta-T Devices, United Kingdom). Dry weight (DW) was weighed after tissue dehydration at +70°C for 48 h (Venticell-BMT, Czech Republic).

### Statistical Analysis

Data were processed using the XLSTAT software (Addinsoft, France), using one-way analysis of variance, ANOVA, and Tukey’s (HSD) test at confidence level *p* = 0.05. Multivariate principal component analysis (PCA) was performed. The results are presented in a PCA scatterplot that indicates distinct levels of metabolites, antioxidant activity, and photosynthetic indices in lettuce subjected to constant or dynamic red and blue light at different photoperiods and a correlation circle (based on Pearson’s correlation matrix) that summarizes the metabolic relations between investigated metabolites, antioxidants, and photosynthetic systems.

## Results

### The Effect of Light Conditions on Leaf Pigments, Soluble Sugars, Photosynthetic Indices and Growth Characteristics

**FIGURE 2:**
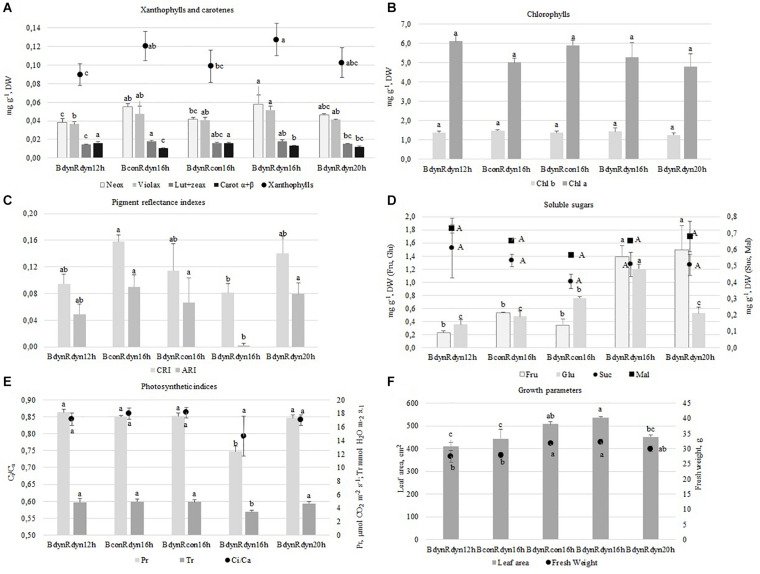
The effect of light conditions on leaf pigments **(A,B,D)**, soluble sugars **(C)**, photosynthetic indices **(E)**, and growth characteristics **(F)** of lettuce. Plants grew under constant (con) or parabolic (dyn) blue (B, 452 nm) and/or red (R, 662 nm) LED PPFD at 12, 16, and 20 h photoperiods, but the same DLI; 2.3 and 9.2 mol m^–2^ per day for B and R. The data were processed using the XLSTAT software and Tukey’s (HSD) test at confidence level *p* = 0.05 (biological replicates, *n* = 5). Neox, neoxanthin; Violax, violaxanthin; Lut + zeax, lutein and zeaxanthin; Carot α + β, α- and β-carotenes; Chl *a*, Chl *b*, chlorophylls *a* and *b*; Fru, fructose; Glu, glucose; Suc, sucrose; Mal, maltose; CRI, carotenoid/chlorophyll ratio; ARI, anthocyanin amount; Pr, photosynthetic rate; Tr, transpiration rate; *C*_*i*_/*C*_*a*_, intercellular to ambient CO_2_ concentration.

A parabolic PPFD profile of 16 h BdynRdyn 16 h resulted in a significant accumulation of Violax (Figure 2A) and hexoses (Fru and Glu) (Figure 2D), along with a significant decrease in pigment reflectance indexes (CRI and ARI) (Figure 2C), *C*_*i*_/*C*_*a*_, Pr, and Tr (Figure 2E). However, the trend of Neox accumulation was similar to constant blue (BconRdyn 16 h) lighting (Figure 1A). The 16 h photoperiod under BdynRdyn and BdynRcon treatments resulted in a significant increase of lettuce fresh weight (11.1%) and leaf area (16.8%) (Figure 2F). However, neither parabolic or constant PPFD patterns nor the duration of photoperiod had a significant effect on the accumulation of chlorophylls (Chl *a*, Chl *b*), sucrose, or maltose (Figures 2B,D). Higher PPFD levels under a 12 h photoperiod (BdynRdyn 12 h) significantly reduced the accumulation of neoxanthin (23.4%), violaxanthin (19.5%), lutein, and zeaxanthin (11.6%). However, the total amounts of xanthophylls were similar to those accumulated under lower PPFD levels under a 20 h photoperiod (BdynRdyn 20 h) or under the constant red (BdynRcon 16 h) PPFD treatment. Constant blue (BconRdyn 16 h) significantly reduced (31.0%), whereas constant red (BdynRcon 16 h) significantly increased (21.9%) the contents of α + β-carotenes (Figure 2A). Under a 12 h photoperiod, hexose accumulated in amounts more than two times lower (0.58 mg g^–1^ DW) than sucrose, compared with other treatments (1.1–2.6 mg g^–1^ DW) (Figure 1D).

### The Effect of Light Conditions on the Variation of Organic Acid Content and Antioxidant Response

**FIGURE 3:**
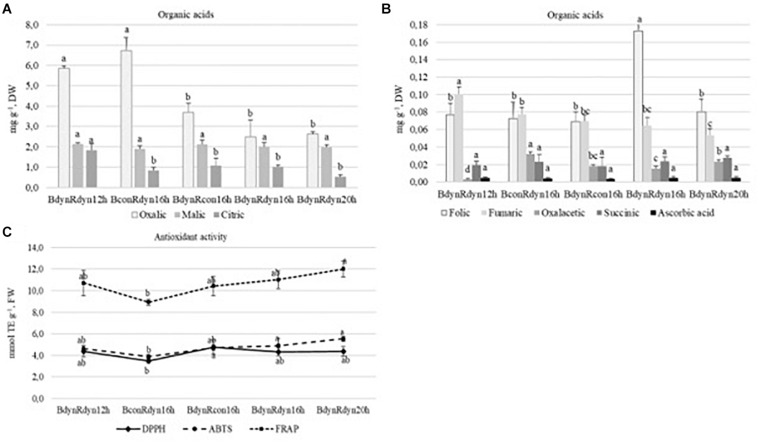
The effect of light conditions on the variation of organic acid **(A,B)** content and antioxidant response with a focus on DPPH, ABTS, and FRAP assays **(C)** of lettuce. Plants grew under constant (con) or parabolic (dyn) blue (B, 452 nm) and/or red (R, 662 nm) LED PPFD at 12, 16, and 20 h photoperiods, but the same DLI; 2.3 and 9.2 mol m^–2^ per day for B and R. The data were processed using the XLSTAT software and Tukey’s (HSD) test at confidence level *p* = 0.05 (biological replicates, *n* = 5).

Substantial amounts of oxalic, malic, and citric acids (7.4–0.5 mg g^–1^ DW) were found in lettuce, with folic and fumaric acids present as a second major group (0.19–0.05 mg g^–1^ DW), while oxaloacetic, succinic, and ascorbic acids were present in the smallest amounts (0.034–0.003 mg g^–1^ DW) (Figures 3A,B). The accumulation of oxalic, citric, and fumaric acids significantly (2.2 times) increased under a 12 h photoperiod. Constant blue (BconRdyn 16 h) resulted in a significant increase in oxalic and oxaloacetic acids (2.2 times both), while parabolic blue (BdynRdyn 16 h) led to a significant increase in folic acid (2.3 times). No significant differences in malic, succinic, or ascorbic acids were found.

The trends of antioxidant activity in response to lighting treatments, based on different assays, were similar (Figure 3C). Constant blue (BconRdyn 16 h) light resulted in the lowest values of DPPH, ABTS^+^, and FRAP (3.5, 3.9, and 8.9 mmol TE g^–1^). Significantly, the highest antioxidant capacity was found under a 20 h photoperiod using the FRAP assay (12.0 mmol TE g^–1^); however, a significant decrease (18.8%) was conditioned only under constant blue (BconRdyn 16 h) treatments. The ABTS^+^ assay showed an antioxidant activity of 5.5 mmol TE g^–1^, whereas the DPPH^∙^ assay showed no significant differences in lettuce extracts compared with the 12- or 16 h parabolic blue and red light profiles (BdynRdyn).

### Light Affected Differences in Metabolic, Antioxidant, and Photosynthetic Response

**FIGURE 4:**
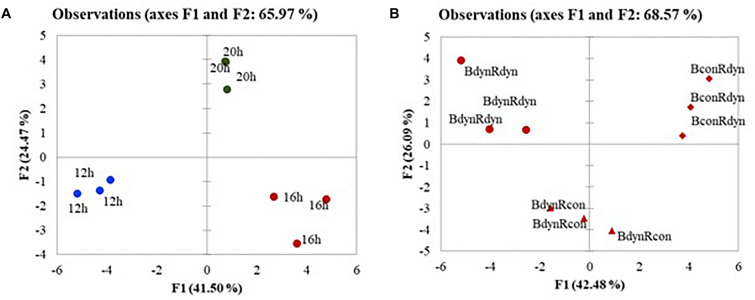
The PCA scatterplot analysis in lettuce, indicating distinct differences in metabolites, antioxidants, and photosynthetic indices depending on lighting conditions in lettuces subjected to parabolic or constant red and blue lighting **(B)** at different photoperiods **(A)**, but similar DLA. Plants grew under constant (con) or parabolic (dyn) blue (B, 452 nm) and/or red (R, 662 nm) LED PPFD at 12, 16, and 20 h photoperiods, but the same DLI; 2.3 and 9.2 mol m^–2^ per day for B and R. The data were processed using the XLSTAT software and Tukey’s (HSD) test at confidence level *p* = 0.05 (biological replicates, *n* = 5).

The PCA score scatterplot (Figure 4) shows the average coordinates of carotenes, xanthophylls, soluble sugars, organic acids, antioxidant activity, and photosynthetic indices in lettuce under constant, parabolic blue and/or red light at 12, 16, and 20 h photoperiods. The first two factors (F1 vs. F2) of the PCA explained 65.97 and 68.57% of the total variance in the photoperiod (Figure 4A) and lighting profile (Figure 4B) responses, respectively. F1 explained approximately 42%, whereas F2 explained 24–26% of the total variability. In terms of the F1 score, the plant responses to parabolic blue and red lighting (BdynRdyn) under the 16 and 20 h photoperiods were clearly distinct from the responses to the 12 h photoperiod (Figure 4A). F1 scores from the 16 h photoperiods (Figure 1B) with parabolic (BdynRdyn) and constant (BconRdyn) blue light treatments were clearly distinct from those with the same photoperiod and parabolic blue and red light treatments.

**FIGURE 5:**
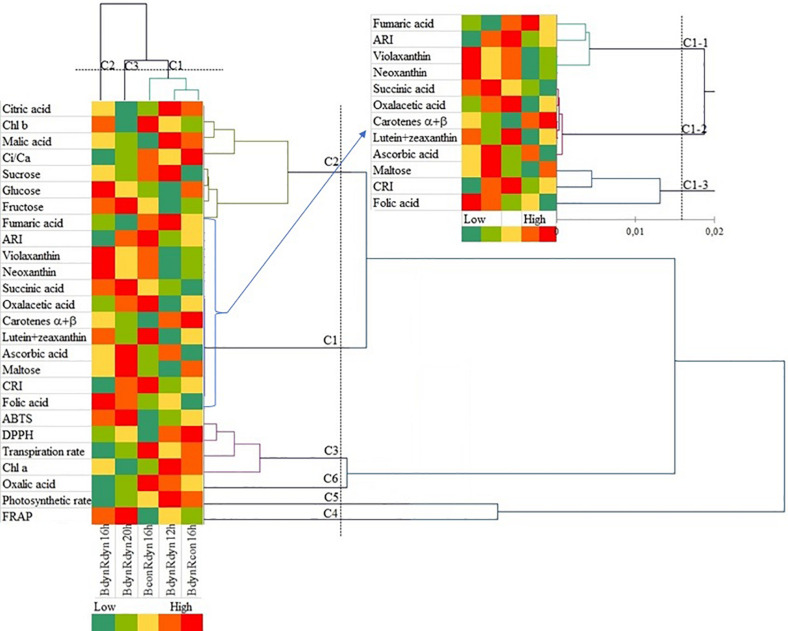
Agglomerative hierarchical cluster (AHC) analysis in lettuces subjected to different light treatments. Pigments, soluble sugars, organic acids, and photosynthetic and antioxidant indices were grouped by similarities in concentration mean and intensity values. Plants grew under constant (con) or parabolic (dyn) blue (B, 452 nm) and/or red (R, 662 nm) LED PPFD at 12, 16, and 20 h photoperiods, but the same DLI; 2.3 and 9.2 mol m^–2^ per day for B and R. The data were processed using the XLSTAT software and Tukey’s (HSD) test at confidence level *p* = 0.05 (biological replicates, *n* = 5).

The agglomerative hierarchical cluster (AHC) analysis was used to divide the responses to the light profile and photoperiod data into groups of increasing dissimilarity (Figure 5). These divisions correspond to the PCA output with the following three clusters: cluster 1 (BconRdyn 16 h, BdynRdyn 12 h, and BdynRcon 16 h), cluster 2 (BdynRdyn 16 h), and cluster 3 (BdynRdyn 20 h). Six clusters of metabolic, photosynthetic, and antioxidant responses were identified. In contrast to cluster 1, clusters 2 and 3, both groups which included parabolic blue and red light profiles (BdynRdyn) at 16 and 20 h photoperiods, were characterized by low *C*_*i*_/*C*_*a*_, Pr, Tr, Chl *a*, DPPH, oxalic, and fumaric acid values and low or medium malic acid, sucrose, and α- and β-carotene content. All analyzed metabolites were in cluster 1 or cluster 2, except for Chl *a* (C3) and oxalic acid (C6). The highest similarity among metabolites was found in cluster 1. Fumaric acid, anthocyanin amount, violaxanthin, and neoxanthin were grouped into cluster C1-1; succinic acid, oxaloacetic acid, carotenes α + β, lutein + zeaxanthin, and ascorbic acid were grouped into cluster C1–2; and maltose, carotenoid/chlorophyll ratio, and folic acid were grouped into cluster C1–3.

A negative correlation between xanthophylls (Neox, Violax, Lut + zeax) and the 12 h photoperiod treatment was found, with the opposite pattern for the 16 h photoperiod treatment. A positive correlation between photosynthetic response (Pr, Tr, *C*_*i*_/*C*_*a*_) and the 12 h photoperiod was found (Table 1). A negative correlation between antioxidant properties (ABTS, FRAP), growth parameters (LA, FW), and the 12 h photoperiod was observed. A positive correlation between LA, FW, and the 16 h photoperiod was found. In contrast to the parabolic blue and red light profiles (BdynRdyn), a positive correlation between Pr, Tr, *C*_*i*_/*C*_*a*_, and constant blue light (BconRdyn) or constant red (BdynRcon) light was observed. A negative correlation between antioxidant properties (DPPH, ABTS, FRAP), growth parameters (LA, FW), and constant blue light (BconRdyn) was found, while a parabolic light profile (BdynRdyn) led to a positive correlation.

**TABLE 1:** Correlation between photosynthetic indices, antioxidant activity, growth parameters, and lighting conditions.

	BdynRdyn			16 h		
	12 h	16 h	20 h	BconRdyn	BdynRcon	BdynRdyn
Neox	−0.673*	0.768*	−0.094	0.277	−0.796*	0.518*
Violax	−0.701*	0.899*	−0.199	0.098	−0.664*	0.567*
Lut + zeax	−0.475*	0.831*	−0.356	0.365	−0.589*	0.223
Chl *a*	0.683*	−0.099	−0.584	−0.513*	0.663*	−0.150
Chl *b*	0.146	0.406*	−0.551	0.309*	−0.334*	0.024
Pr	0.600*	−0.973*	0.373	0.470*	0.513*	−0.982*
Tr	0.544*	−0.878*	0.335	0.478*	0.458*	−0.935*
*C*_*i*_/*C*_*a*_	0.329*	−0.611*	0.282	0.344*	0.391*	−0.734*
DPPH	0.019	−0.022	0.003	−0.772*	0.622*	0.150
ABTS	−0.538*	−0.163	0.701	−0.782*	0.277	0.505*
FRAP	−0.403*	−0.163	0.566	−0.798*	0.195	0.603*
LA	−0.742*	0.927*	−0.184	−0.838*	0.184	0.654*
FW	−0.756*	0.739*	0.017	−0.944*	0.389	0.555*

*Correlation coefficient (r) for the pairs of investigated parameters (n = 3 for each parameter) is presented. Correlations marked with asterisk mark (^∗^) are significant at p < 0.05. B, blue, 452 nm; R, red, 662 nm; dyn, parabolic profile; con, constant profile; Neox, neoxanthin; Violax, violaxanthin; Lut + zeax, lutein and zeaxanthin; Chl a, Chl b, chlorophylls a and b; Pr, photosynthetic rate; Tr, transpiration rate; C_i_/C_a_, intercellular to ambient CO_2_ concentration; LA, leaf area; FW, fresh weight.*

A positive correlation was found between xanthophylls (Neox, Violax, Lut + zeax) and photoperiod, with the content of these compounds increasing as the length of the light cycle increased (12–16 h). The opposite pattern was observed between photosynthetic response (Pr, Tr, *C*_*i*_/*C*_*a*_) and photoperiod (Table 1). Similarly, antioxidant properties (ABTS, FRAP) and growth parameters (LA, FW) increased with increasing photoperiod (12–16 h). Pr, Tr, and *C*_*i*_/*C*_*a*_ increased under the constant blue (BconRdyn) or constant red (BdynRcon) light treatments compared with the other light treatments. The same was true for antioxidant properties (DPPH, ABTS, FRAP) and growth parameters (LA, FW) under the parabolic light profile BdynRdyn, with the opposite responses observed for treatments with the constant blue (BconRdyn) light profile.

## Discussion

### Photosynthetic Response and Primary Metabolism

When plants acclimate to changing light conditions, physiological responses are the first to occur, leading to morphological, photosynthetic, and metabolic changes. Detailed knowledge about the photosynthetic pigment pool is critical for understanding the light-harvesting mechanism and photoacclimation potential of plants. The modulation and balance of photosynthetic pigment contents are part of the photosynthesis process and contribute to attaining proper equilibrium between the energy input and output (Ruban et al., 2011). Changing the photoperiod under parabolic red and blue light profiles (BdynRdyn) had a significant effect on the accumulation of neoxanthin and violaxanthin. The 12 and 16 h photoperiods led to a significant decrease and increase, respectively, in the xanthophyll content (Figure 2A). Although chlorophylls are the main light-capturing antennae pigments, changes in neoxanthin and violaxanthin ratio should act as a signal for light-harvesting antennae efficiency, finally resulting in acclimations to the lighting environment and lower tendencies to switch to the photoprotective mode (Gruszecki, 2010). No significant differences among chlorophylls *a* and *b*, lutein, and zeaxanthin content were found. However, parabolic blue and red light under a 16 h photoperiod led to a significant decrease in the carotenoid to chlorophyll ratio (CRI). The trend of the response of carotenes and chlorophylls to all lighting conditions in this study was the same (Figures 2A–C). Thus, it can be presumed that under parabolic red and blue light profiles, the xanthophyll cycle pigments may lead to dissipation of excess energy, with carotenoids concurrently dissipating the excitation energy from chlorophyll, limiting the possible light damage to membranes.

Accumulation of soluble carbohydrates is associated with decreased photosynthesis and increased photoinhibition in the leaves (Urban and Alphonsout, 2007), and this negative feedback effect was also observed in the present study (Figures 2D,E). Apel and Hirt (2004) suggested that the inhibiting effect of soluble sugar accumulation on the Calvin cycle leads to the formation of singlet oxygen in PS II. Fanciullino et al. (2014) supposed that glucose stimulates the synthesis of carotenoids, especially xanthophylls, which are involved in the protection of photosystems against photooxidative stress. However, AHC analysis did not show any direct connections between glucose (cluster C2) and carotenoids (cluster C1) (Figure 5). It is known that glucose and fructose are primary, while sucrose and starch are the major end products of photosynthetic activity in most plants. The process of sucrose and starch formation does not depend on light because sucrose can be synthesized from glucose and fructose in the dark and without chlorophyll (Stein and Granot, 2019). We also obtained similar results; neither sucrose nor maltose was affected by different light profiles or photoperiod (Figure 2D), indicating that the synthesis of disaccharides is a process independent from photosynthesis. Meanwhile, AHC analysis showed a similar trend of sucrose accumulation and *C*_*i*_/*C*_*a*_, which was opposite to the trend of fructose and glucose accumulation (Figure 5). A strong negative correlation between *C*_*i*_/*C*_*a*_ and parabolic blue and red light under a 16 h photoperiod was found, while other light profiles or durations resulted in a weak correlation (Table 1). Thus, the stomatal behavior regulation might depend on both external environmental and internal signaling cues (Lawson and Blatt, 2014). Annunziata et al. (2017) found that the accumulation of starch in *Arabidopsis* grown under artificial light with a sinusoidal light profile was more intensive at the end of the day compared with that grown under constant irradiance or natural light. Parabolic lighting (BdynRdyn 16 h) led to a significant increase in fructose and glucose (Figure 2D), compared with other lighting treatments, suggesting that plants avoid C starvation before photosynthesis starts after the dark period (Stitt and Zeeman, 2012). However, the decrease in *C*_*i*_/*C*_*a*_ resulted in a significant decrease in photosynthetic and transpiration rates (Figure 2) under a parabolic blue and red lighting treatment with a 16 h photoperiod (Figure 2E). McAusland et al. (2013) suggested that stomatal responses tend to be slower than photosynthetic responses, leading to suboptimal responses in stomatal conductance and photosynthetic rate, resulting in lower carbon gain. The decrease in photosynthetic rate and stomatal conductance observed over longer time scales, related to red light changes toward the end of the day, may be explained by sugar import into guard cells (Matthews et al., 2020). Sucrose transported to the guard cells is cleaved in the apoplasts to produce hexoses, which are sensed by hexokinase, which in turn signals for the stomatal closure response (Kelly et al., 2013). In contrast to that for red light, the stomatal blue light response, which has been reported to not require photosynthesis (Hiyama et al., 2017), is important for stomatal opening in the morning, when the irradiance spectrum is steeped in blue wavelengths. These findings suggest that light is only required for the reactions involving the primary phases of CO_2_ utilization during photosynthesis. Chen et al. (2019) found that a dynamic red/blue light environment had no positive effects on glucose and fructose accumulation in lettuce, in contrast to its effect on sucrose, and they also found that monochromatic lighting had the opposite effect. Our results show that hexoses were more sensitive to the lighting profile than sucrose or maltose (Figure 2D). These differences may be due to differences in DLI: the DLI was maintained at 11.54 mol m^–2^ per day in the current study, whereas Chen et al. (2019) used 7.49 mol m^–2^ per day. The highest total soluble sugar content under parabolic blue and red light was found in the 16 h photoperiod (BdynRdyn 16 h) treatment, which showed a significantly higher fresh weight and new leaf area formation (Figures 2D,F). Other results have shown that alternating red and blue light at intervals resulted in better yield and taste. Moreover, the duration of the interval between red and blue light largely influenced the dynamics between the signal and response pathways (Chen et al., 2019).

### Antioxidant Response and Secondary Metabolism

Environmental fluctuations and external stress are the main factors enhancing the antioxidant response. While most studies have indicated that red and blue components are more effective against antioxidant responses, these treatments were performed on *in vitro* callus cultures and only constant monochromatic spectral lights were investigated (Nazir et al., 2020; Usman et al., 2020). Nam et al. (2018) showed that blue light, compared with red or fluorescent, could be applied to enhance flavonoid levels and antioxidant activity in common buckwheat sprouts. A significant increase in antioxidant activity, based on the DPPH assay, was found in microgreens, such as red pak choi and basil, under 25 and 33% of constant blue light (Vaštakaitė et al., 2015). Son and Oh (2013) also stated that the antioxidant capacity (expressed as TEAC) of both red and green leaf lettuces grown under high ratios of blue LED (such as 59, 47, and 35% B) was significantly higher than the lower blue to red ratio. Antioxidant activity in response to parabolic or constant light profiles was evaluated by employing antioxidant assays, mainly DPPH, ABTS, and FRAP assays. Compared with the other treatments, the lowest antioxidant activity of all the assays was recorded under constant blue and parabolic red light treatments with the 16 h photoperiod (Figure 3C). The increase in antioxidant capacity and induced accumulation of phenolic compounds under additional irradiation of blue LEDs in combination with red light was explained by the decreased growth of lettuce (Stutte et al., 2009; Johkan et al., 2010; Son and Oh, 2013). However, such tendencies were not observed among changes in lettuce fresh weight or leaf area (Figure 2F) and antioxidant activity (Figure 3C) in the present study.

The reason for the apparent C deficiency, or more specifically, the deficiency in organic acids, in plants grown under natural light is unclear (Annunziata et al., 2017). Plants adapted their C metabolism in response to sunny and cloudy days balancing their C and N metabolism. Sucrose signaling metabolites have been implicated in C/N interactions through activation of phosphoenolpyruvate carboxylase and nitrate reductase and increased anaplerotic flux of C into organic acids (Figueroa et al., 2016). However, sucrose did not differ significantly (Figure 2D) between lighting treatments; moreover, AHC analysis did not show any similarities with organic acids, except for malic and citric acids (Figure 5). The changes in organic acid content (Figures 3A,B) suggest that metabolism of organic acids may be less robust to changes in light intensity profiles than to photoperiod. Ascorbic acid is involved in many plant physiological processes, including photosynthesis or transmembrane electron transport (Plumb et al., 2018). In agreement, AHC analysis showed close similarity among ascorbic acid, lutein, zeaxanthin, and carotenes (cluster C1–2) (Figure 5). On the other hand, neither parabolic nor constant lighting profiles or different photoperiods (it should be stated that DLI was the same in all treatments) had a significant effect on ascorbic acid accumulation (Figure 3B). A significant increase in ascorbic acid was mainly obtained by extending the continuous light for 48 (Zhou et al., 2012) or 72 h (Yabuta et al., 2007). Moreover, in agreement with Yabuta et al. (2007), the dissimilarity among hexoses, sucrose (cluster C2), and ascorbic acid (cluster C1) (Figure 5) shows that photosynthetic electron transport in chloroplasts is highly related to ascorbic acid pool size regulation in the leaves but is independent of sugars.

## Conclusion

The results obtained and the PCA analysis confirmed the significant impact of both photoperiod and the parabolic profiles of PPFD distribution on lettuce physiological response. A 16 h photoperiod resulted in significantly higher xanthophyll content (neoxanthin, violaxanthin, lutein, and zeaxanthin) in lettuce leaves under both constant and parabolic blue light treatments (BconRdyn 16 h; BdynRdyn 16 h). Lower and higher PPFD levels under a 20 and 12 h photoperiod (BdynRdyn 20 h, BdynRdyn 12 h), respectively, and maintaining the same DLI had a pronounced reducing impact on photosynthetic indices (Pr, Tr, *C*_*i*_/*C*_*a*_), xanthophylls, soluble sugar contents, and antioxidant properties of lettuce leaves. Parabolic lighting (BdynRdyn 16 h) led to a significant decrease in *C*_*i*_/*C*_*a*_, resulting in decreased photosynthetic and transpiration rates, compared with constant blue or red light PPFD over the same photoperiod (BconRdyn, BdynRcon at 16 h). Moreover, under constant blue lighting, higher carotenes α + β, anthocyanin (ARI) content, and CRI were obtained, but these were accompanied by decreased biomass accumulation and antioxidant activity.

## Data Availability Statement

The original contributions presented in the study are included in the article/supplementary material, further inquiries can be directed to the corresponding author/s.

## Author Contributions

GS: data analysis, writing of the manuscript. AV: joint coordination of the experiment, modeling of light parameters, and data summarizing JM: spectrophotometric analysis, biometric measurements, and data summarizing. PH: chromatographic analysis. KL: photosynthesis and optical indices measurements, data summarizing. AB and PD: the realization of lighting schedules in vegetative experiments, data analysis All authors read and approved the final version of the manuscript.

## Conflict of Interest

The authors declare that the research was conducted in the absence of any commercial or financial relationships that could be construed as a potential conflict of interest.
